# Skeletal Muscle Quality: A Biomarker for Assessing Physical Performance Capabilities in Young Populations

**DOI:** 10.3389/fphys.2021.706699

**Published:** 2021-08-05

**Authors:** Marshall A. Naimo, Alyssa N. Varanoske, Julie M. Hughes, Stefan M. Pasiakos

**Affiliations:** ^1^Military Performance Division, United States Army Research Institute of Environmental Medicine, Natick, MA, United States; ^2^Military Nutrition Division, United States Army Research Institute of Environmental Medicine, Natick, MA, United States; ^3^Oak Ridge Institute for Science and Education, Oak Ridge, TN, United States

**Keywords:** performance, high-intensity exercise, adaptation, muscle architecture, stretch-shortening contractions, cross-sectional area, ultrasound

## Abstract

Muscle quality (MQ), defined as the amount of strength and/or power per unit of muscle mass, is a novel index of functional capacity that is increasingly relied upon as a critical biomarker of muscle health in low functioning aging and pathophysiological adult populations. Understanding the phenotypical attributes of MQ and how to use it as an assessment tool to explore the efficacy of resistance exercise training interventions that prioritize functional enhancement over increases in muscle size may have implications for populations beyond compromised adults, including healthy young adults who routinely perform physically demanding tasks for competitive or occupational purposes. However, MQ has received far less attention in healthy young populations than it has in compromised adults. Researchers and practitioners continue to rely upon static measures of lean mass or isolated measures of strength and power, rather than using MQ, to assess integrated functional responses to resistance exercise training and physical stress. Therefore, this review will critically examine MQ and the evidence base to establish this metric as a practical and important biomarker for functional capacity and performance in healthy, young populations. Interventions that enhance MQ, such as high-intensity stretch shortening contraction resistance exercise training, will be highlighted. Finally, we will explore the potential to leverage MQ as a practical assessment tool to evaluate function and enhance performance in young populations in non-traditional research settings.

## Introduction

The ability of skeletal muscle to produce substantial force in relatively short periods of time is crucial to success in many sporting events, tactical operations, and activities of daily living. While the focus of improving muscular force generation traditionally centers on increasing muscle quantity (i.e., muscle hypertrophy), recent efforts have focused on improving muscle quality (MQ) for enhancing muscle function. MQ is an umbrella term referring to the ability of skeletal muscle to perform several functions effectively, including force production, contraction and relaxation, metabolism, substrate turnover and storage, heat generation, myokine production, and electrical conduction (Fragala et al., 2015; Correa-de-Araujo et al., 2017). Although no consensus definition of MQ exists as of yet, the ambiguity of the term has allowed investigators to examine several dimensions of MQ that relate to muscle function. As the most prominent function of skeletal muscle is force production, the present review will focus on MQ in this context; however, the role of skeletal muscle in other biological functions should not be overlooked.

In applied and clinical research settings, MQ is often quantified as muscle strength or power per unit of muscle mass (Barbat-Artigas et al., 2012; McGregor et al., 2014), commonly reported as peak force or maximal power production relative to a measure of muscle size, such as muscle volume or cross-sectional area (CSA). This definition accounts for the functional significance of the architecture, the overall tissue characteristics of the skeletal muscle, and ultimately, force production capabilities (Mangine et al., 2020). Increased force output as a product of increased muscle mass is a well-established physiological concept (Bamman et al., 2000); however, research demonstrates that force generation is largely a product of skeletal MQ aside from muscle quantity (Ivey et al., 2000; Brooks et al., 2006; Goodpaster et al., 2006; Fukumoto et al., 2012). The distinction between MQ and muscle quantity is an important aspect of functional assessment, as two individuals with similar muscle mass may not be capable of producing equivalent amounts of force. Similarly and counterintuitively, individuals with smaller amounts of muscle mass may be more effective at producing force than those with greater amounts. Differences in MQ between individuals may help explain why certain muscles are stronger than others, despite similar sizes. However, MQ may also vary between limbs, generating potential for injury if not properly corrected.

Muscle quality has largely been studied in aging and adult populations with pathophysiological conditions, and in those studies, higher MQ is related to increased muscle strength, function, and physical performance, and vice versa (Fragala et al., 2015; Brown et al., 2016). As posited in this review, understanding the phenotypic attributes of MQ and using MQ as an assessment tool to explore the efficacy of exercise training that prioritizes functional enhancement over increases in muscle size may have implications for populations beyond compromised adults, including healthy young adults who routinely perform physically demanding tasks for competitive or occupational purposes. Accordingly, this review will critically examine MQ and the evidence base that supports MQ as a practical and important biomarker for functional capacity and performance in healthy, young populations. Interventions that augment MQ, such as high-intensity stretch shortening contraction resistance exercise training (RET), will be highlighted. Finally, we will explore the potential to leverage MQ as a practical assessment tool to evaluate function and performance enhancement in young populations in non-traditional research settings.

## Discerning MQ From Muscle Size Alone

One of the most important features of muscle is that it is dynamically regulated by changes in physical demands, as well as environmental challenges, and is remarkably plastic in response to various forms of stimuli (Caldow et al., 2015). One of the defining characteristics of exercise is its ability to initiate a complex assortment of highly coordinated molecular and cellular signaling events, as well as transcriptional regulators, which in turn have profound positive effects on muscle tissue (Bamman et al., 2014). Upon exposure to physical activity demands, muscle responds in a coordinated fashion by initiating various signaling events that drive changes in gene expression of intracellular processes, such as metabolism, alterations in hormone signaling, defense mechanisms against oxidative stress, DNA damage and repair, protein turnover, and muscle remodeling (Phillips et al., 2013; Camera et al., 2016). Thus, customary stimuli by appropriate activity or exercise prompt changes in gene expression, which ultimately results in an integrated physiological and functional adaptation in skeletal muscle and the surrounding soft tissue. Therefore, by solely examining changes in muscle size with exercise, the true exquisiteness of skeletal muscle functional adaptation is lost.

While the relationship between increased muscle size and increased force output is well-established, force generation is a result of several factors aside from exclusively mass (Ivey et al., 2000; Goodpaster et al., 2006; Fukumoto et al., 2012). The relationship between size and strength is not linear, as strength can be improved independently of significant increases in muscle growth (Parente et al., 2008; Dankel et al., 2017; Mattocks et al., 2017), and likewise, increases in size can occur without concomitant increases in force production (Ahtiainen et al., 2016; Jakobsgaard et al., 2018). Thus, beyond muscle size, other factors such as tissue composition (e.g., contractile and non-contractile components), architecture, metabolism, and neural activation contribute to the overall force production capabilities (McGregor et al., 2014). For example, lower-body CSA and volume are associated with greater adiposity (Varanoske et al., 2017), indicating that increased intramuscular fat may account for greater muscle volume. However, in this case, greater muscle size will likely not translate to greater force production. Factors contributing to muscle strength and size are listed in Table 1.

**TABLE 1:** Factors contributing to increased muscle strength and size, which are important determinants of MQ.

Factors contributing to increased muscle strength	Factors contributing to increased muscle size
• Fiber type distribution	• ↑ Contractile tissue
•↑ Type II: Type I fibers	•↑ Myofibrillar hypertrophy
• Muscle architecture	•↑ Sarcoplasmic hypertrophy
•↑ Pennation angle	• ↑ Fibrous/connective tissue
•↓ Fascicle length	• ↑ Intramuscular/Intermuscular/Intramyocellular fat
• ↑ Contractile tissue	• ↑ Edema/Swelling
•↑ Myofibrillar hypertrophy	
•↔ Sarcoplasmic hypertrophy	
• ↓ Fibrous/Connective tissue	
• ↓ Intramuscular/Intermuscular/Intramyocellular fat	
• Neuromuscular activation	
•↑ Motor unit firing rate	
•↑ Motor unit synchronization and recruitment	
•↑ Neural drive	
•↑ Disinhibition of autogenic inhibitory mechanisms	
•↓ Muscle antagonist activation	

In this regard, muscle architectural characteristics that affect force production may change following RET, leading to improved muscle strength, power, and subsequently overall MQ. For instance, increases in fascicle length and pennation angle have been observed following RET (Kawakami et al., 1995; Aagaard et al., 2001; Blazevich et al., 2007; Seynnes et al., 2007; Baroni et al., 2013; Ema et al., 2013; Franchi et al., 2014, 2016; McMahon et al., 2014), with RET-induced increases in pennation angle implied to be associated with increased force production attributable to the degree of muscle hypertrophy experienced (Azizi and Brainerd, 2007; Ema et al., 2013). Interestingly, increases in fascicle length do not always accompany significant muscle hypertrophy (Fukutani and Kurihara, 2015), demonstrating that muscle force production is greater than just a factor of its size. Thus, measures of size alone, such as muscle CSA, inadequately estimate force production. This is why in recent years, sarcopenia has evolved from being defined exclusively as the age-related loss of muscle mass to more contemporary definitions that account for loss of strength or physical performance (Cruz-Jentoft et al., 2019). However, this same line of thinking has yet to be applied to younger populations, including research, clinical, and standard occupational assessment settings, as measures of body composition, muscle mass, and/or static strength continue to be prevalent in these environments.

Relying exclusively on measures of strength (e.g., handgrip), size (e.g., CSA), or performance alone likely limits our ability to draw meaningful conclusions about the effectiveness of a training intervention because they do not tell a complete story. For instance, military body fat standards, which emphasize abdominal circumference measures and/or bioelectrical impedance analysis of body composition, not only have their own flaws when it comes to accuracy and reliability, but are also outdated because recruits today tend to have more body fat and more lean mass compared to previous generations, which aids in their ability to perform lifting and carrying tasks (Foulis et al., 2020). Moreover, there is an increased prevalence of those soldiers who are metabolically obese but normal weight, with low muscle mass and thus overall poor function (Foulis et al., 2020). Absolute handgrip strength is often used in clinical and research settings as a measure of muscle strength and function, but research has demonstrated that this is not the best indicator of function and that forearm MQ is a stronger predictor of physical performance (Abe et al., 2016). Because the majority of physically demanding tasks performed by younger individuals (e.g., load carriage during military operations) requires a combination of muscle strength, power, and size in order to maximize the potential of these individuals to perform their duties (Szivak, 2020), MQ should be viewed as a critically important metric for evaluating the capabilities of these individuals to maintain effective performance and reduce the likelihood of injury prior to deployment.

## Assessment of MQ

Measures of muscle size and function are often integrated into a single measure of MQ, termed relative strength (Correa-de-Araujo et al., 2017), which is more informative for laboratory or field studies when findings demonstrate improvements or declines in performance in response to a given stressor, or changes in muscle mass without associated changes in function. Relative strength can be calculated by measuring the strength or power of a muscle/muscle group divided by muscle mass. However, several methodologies of measuring muscle size and strength exist, resulting in limited consensus on the most appropriate way of assessing MQ. For instance, physical movement is often the result of several muscles working in synchrony to produce a desired effect, and thus, it becomes difficult to isolate the strength of an individual muscle. Additionally, differences in the type of exercise assessment administered (e.g., isometric, isokinetic, isotonic, dynamic, single-joint, and multi-joint, etc.) will result in different strength values, diminishing the predictive ability of relative strength measures. In this regard, Wheeler et al. (2014) used a specialized statistical approach and applied it to studies previously conducted in young and old rats that were subjected to a previous animal RET protocol (Cutlip et al., 2006; Murlasits et al., 2006; Baker et al., 2010) and demonstrated that dynamic forces were more robust and specific for representing the occurrence of skeletal muscle adaptation compared to isometric values.

The examination of muscle composition may provide additional insight into MQ which can circumvent some of the limitations associated with muscle strength measures. The Bergstrom needle biopsy technique (Bergstrom, 1975) provides useful information about fiber type distribution, muscle capillarization, intramuscular fat content, and muscle metabolism, which aid in predicting muscle function; however, invasive muscle biopsies may not be appropriate for routine MQ assessment. Advancements in fine needle biopsies techniques allow for a less-invasive immunohistological and morphological analysis of muscle tissue (Townsend et al., 2016), but this method still requires repeated muscle sample collection, which may not be practical for most populations. In contrast, muscle imaging techniques, such as computed tomography (CT), peripheral quantitative computed tomography (pQCT) magnetic resonance imaging (MRI), and ultrasonography (US) serve as non-invasive approaches for assessing muscle size and tissue composition, allowing for surrogate measures of MQ that can be derived independently of muscle strength measures. Previous studies have reported skeletal muscle-based outcome measures using dual x-ray absorptiometry (DXA) in various healthy populations following RET despite the notion that DXA cannot distinguish between muscle mass and MQ (Heymsfield et al., 2015; Phillips et al., 2017). Anthropometric measurements that are commonly used to represent skeletal muscle health, such as skinfolds and circumferences, not only have their own inherent limitations such as poor precision/reproducibility, they provide no information pertaining to the underlying contractile and non-contractile tissue components that influence functional outcomes and account for disconnects between size and force production (Heymsfield et al., 2015). Therefore, muscle imaging may be preferred for MQ assessment when muscle strength measures cannot be obtained or when muscle composition estimates are also desired, as relative strength measures do not provide any information on muscle composition. However, the reliability of MQ measures are dependent on the method of examination. For example, the reliability of echo intensity measured via ultrasonography will be different from that of radiological density measured by MRI, which will be different from that of relative strength. Nevertheless, all methods of assessing MQ mentioned herein have been reported to be reliable across different populations and are widely cited throughout the literature. Advantages and disadvantages of these imaging techniques used to measure MQ are presented in Table 2.

**TABLE 2:** Imaging methods of assessing MQ commonly reported in the literature.

Method	Measurements pertaining to MQ	Advantages	Disadvantages	References
Magnetic resonance imaging/spectroscopy (MRI/MRS)	Radiological density	Gold standard for measuring muscle size and cross-sectional area; provides high-resolution images of tissues with a great level of contrast between tissue types, making visualization of superficial, and deep tissues easy; capable of differentiating between intramuscular and intermuscular fat with high quality visualization of intramuscular fat deposition and has a higher sensitivity for detecting myosteatosis compared with CT along with better visualization of anatomical features; does not emit radiation; can differentiate between edema, fatty infiltration, and fibrosis; MRS permits the distinction between intramyocellular lipids and extramyocellular lipids by measuring concentrations of chemical compounds within tissues	Large apparatus; expensive; not readily accessible; cannot be used in patients with metal implants; scans are time-consuming; subject size limitations	Miljkovic and Zmuda, 2010; Gloor et al., 2011; Pillen and van Alfen, 2011; Ahtiainen et al., 2016; Erlandson et al., 2016
Computerized Tomography (CT)	Radiological density	One of the most of reliable and accurate methods of assessing muscle mass and myosteatosis, especially in clinical settings; produces high-resolution, cross-sectional images of body “slices,” which can be combined to estimate muscle volumes and whole body muscle mass; high correlation between muscle lipid content assessed via muscle biopsy and muscle radiological density by CT	Large apparatus; expensive; not readily accessible; unable to distinguish fibrotic tissue from normal muscle except in myosteatosis or edema (cannot provide MQ past fat infiltration); unable to isolate individual muscle groups or distinguish the location of intramuscular fat; radiation exposure; scans are time-consuming	Goodpaster, 2002; Machann et al., 2003; Larson-Meyer et al., 2006; Pillen and van Alfen, 2011; Heymsfield et al., 2015; Correa-de-Araujo et al., 2020
Peripheral quantitative computerized tomography (pQCT)	Radiological density	Similar advantages to CT but with lower cost, increased portability, smaller size, shorter scan time, and lower emittance of radiation in comparison to CT	Similar limitations to CT (cannot isolate individual muscle groups and cannot determine the location of intramuscular fat, emits radiation); cannot be used to scan axial anatomical features due to its smaller size, so images of the lower leg are most commonly reported	Erlandson et al., 2016, 2017; Correa-de-Araujo et al., 2020
Ultrasound (US)	Echo intensity	Low cost, portability, easy to use, and available in both clinical and research settings; does not emit radiation; echo intensity values are significantly related to intramuscular fat and fibrous tissue content, radiological density measures derived by CT, muscle strength, power, and function across the population spectrum; can obtain measures of muscle architecture, including fascicle length and pennation angle, as well as muscle cross-sectional area and thickness; developments in 3-dimensional musculoskeletal US have allowed for quantification of muscle volume; dynamic ultrasound imaging, in which consecutive images are captured during physical movement, permits measurement of muscle contraction characteristics that are absent from static measures; US elastography measures can be used to determine skeletal muscle stress, strain, and elastic modulus	Requires extensive operator experience and prone to large measurement error; EI affected by US settings and systems, muscle thickness, subcutaneous fat thickness, patient positioning, rest time, joint angle, proximity to exercise, hydration status, measurement site, etc.; limited to examining only small portions of the muscle at once	Reimers et al., 1993; Pillen et al., 2009; Ihnatsenka and Boezaart, 2010; Cadore et al., 2012; Drakonaki et al., 2012; Radaelli et al., 2012; Jajtner et al., 2013, 2015; Fragala et al., 2014a; Sikdar et al., 2014; Mangine et al., 2015a; Young et al., 2015; Hacker et al., 2016; Arroyo et al., 2018; Watanabe et al., 2018; Varanoske et al., 2019, 2020

CT produces high-resolution, cross-sectional images of body “slices,” which can be combined to estimate muscle volumes and whole body muscle mass (Heymsfield et al., 2015; Correa-de-Araujo et al., 2020). However, CT is unable to distinguish fibrotic tissue from normal muscle tissue, limiting the ability of CT to provide measures of MQ that exclude impacts of fat infiltration. CT is also unable to isolate individual muscle groups or distinguish the location of intramuscular fat. (Goodpaster, 2002; Machann et al., 2003). Advancements in CT technology have resulted in the development of pQCT machines, which enable 3-dimensional CT scans of the peripheral skeleton. Advantages of pQCT over CT include its lower cost, increased portability and smaller size, shorter scan time, and lower emittance of radiation (Erlandson et al., 2016, 2017). However, pQCT shares some of the same limitations in that it is incapable of isolating individual muscle groups and cannot determine the location of intramuscular fat. MRI is often considered the gold standard method for measuring muscle size and CSA (Ahtiainen et al., 2010) and provides high-resolution images of body tissues, making visualization easy (Pillen and van Alfen, 2011).

MRI is also more sensitive than CT for detecting muscle fat infiltration and visualizing anatomical features (Gloor et al., 2011). Furthermore, MRI assesses the detailed structure and composition of individual muscles and differentiates between edema, fatty infiltration, and fibrosis (Erlandson et al., 2016).

While there is currently no gold standard imaging device to assess MQ, the use of US has become increasingly prevalent in recent research due to the low cost, safety, portability, and reliability as an imaging device. US has recently emerged as one of the most popular ways to assess muscle morphology; like MRI, US does not emit radiation, making it attractive for repeated use. US-derived measures of muscle CSA have demonstrated high reliability and accuracy in comparison to other imaging techniques (Ahtiainen et al., 2010; Noorkoiv et al., 2010; Scott et al., 2012). MQ measures are based off of echo intensity (EI), or brightness, of the tissue in the US image. Thus, muscle with a greater proportion of non-contractile tissue appears brighter in color, accompanied by a higher EI value, and ultimately lower MQ. Several studies report that EI is related to muscle strength, power, and function across the population spectrum, demonstrating that EI may be a proxy measure of MQ (Jajtner et al., 2013; Watanabe et al., 2013; Fragala et al., 2014b; Mangine et al., 2015a; Stock and Thompson, 2020; Wong et al., 2020).

## Exercise and MQ

In general, exercise training elicits positive adaptations to MQ. However, as outlined in Table 3, in comparison to other exercise training regimens such as aerobic exercise training or hypertrophy-based moderate intensity resistance exercise, high-intensity RET appears to be the primary intervention for improving force production, power development, and subsequently augmenting MQ as well as overall muscle function, especially when consisting of stretch-shortening contractions (SSCs) (Fry, 2004; Rader and Baker, 2017; Rader et al., 2018a; Szivak, 2020; Duchateau et al., 2021). SSCs consist of sequential isometric, eccentric, and concentric contractions, such as the squat, during which the muscle is activated prior to (isometric - this represents the stabilization phase when holding the bar prior to the descent) as well as during the initial stretch/lengthening (eccentric- the descending portion of the squat) and subsequent shortening (concentric- the ascending portion of the squat) (Cutlip et al., 2006; Rader and Baker, 2017; Rader et al., 2017b). SSCs provide a significant advantage over isolated isometric-, concentric-, or eccentric-only contractions because they are more physiologically relevant as the typical contraction sequence utilized by mammals that occur during activities of daily living as well as the majority of exercise movements (e.g., walking, sprinting, resistance training, and plyometrics) (Vaczi et al., 2014; Rader and Baker, 2017, 2020; Rader et al., 2018a). As most traditional free-weight RET paradigms incorporate SSCs as part of the typical routine (i.e., most training programs do not isolate a single type of contraction), we will refer to RET using SSCs as “RET” for the remainder of the paper. Common physiological adaptations promoted specifically by a high-intensity RET stimulus (≥70% one-repetition maximum, 1RM) include an augmentation of muscle capillarization, reduced muscle lipid content, increased motor unit recruitment, and a selective increase in the number of type II muscle fibers that are activated during training, which are able to generate the greatest force output and have the highest capability for growth (Frontera et al., 1988; Hepple et al., 1997; Hagerman et al., 2000; Vaczi et al., 2014; Rader and Baker, 2017; Szivak, 2020). While increases in muscle mass have been reported to occur over a broader range of intensities (American College of Sports Medicine, 2009; Shoepe et al., 2020), preferential increases in type II muscle fiber CSA have been shown specifically through high-intensity training protocols (Fry, 2004; Vann et al., 2020).

**TABLE 3:** Different training modalities for improving MQ in young, healthy populations.

Type of exercise	Advantages	Disadvantages	References
**Aerobic - Low to moderate intensity (continuous endurance)**	Shown to improve muscle capillarization and cardiorespiratory fitness; significantly decreases adipose tissue, subsequently improving lean mass to adipose ratio and overall tissue quality	Can lead to decreased muscle mass; been shown to be less optimal for improving strength and power output compared to HIIT and RET; concurrent training (i.e., simultaneous inclusion of endurance and resistance training) has been shown to lead to impairments in explosive strength of trained muscles	Putman et al., 2004; Wilson et al., 2012; Hellsten and Nyberg, 2015; Hughes et al., 2018; Petre et al., 2021
**Aerobic - HIIT**	Demonstrated to produce greater improvements in muscle exercise capacity, anaerobic power, and mitochondrial biogenesis compared to traditional continuous endurance exercise; shown positive effects on muscle structure, muscle mass, and body composition; in contrast to continuous endurance training, concurrent HIIT with RET appears to be less detrimental for inhibiting gains in muscle mass and upper body strength; shown to have positive effects on muscle power and field test performance	No evidence that HIIT aerobic exercise improves muscle strength; magnitude of positive effects on muscle mass are not as great as with traditional RET; been shown that when performed following RET, HIIT may decrease RFD as a consequence to changes in muscle architecture	Tanisho and Hirakawa, 2009; Ziemann et al., 2011; MacInnis and Gibala, 2017; Tsitkanou et al., 2017; Martland et al., 2020; Vechin et al., 2021
**Low- to moderate-intensity RET**	Shown to lead to improvements in muscle strength, power, and, specifically at moderate intensities, increases in muscle mass; results have shown improvements in performance	Inferior to higher intensities for increasing muscle strength and improving performance; evidence for moderate intensity being better than high-intensity RET for increasing muscle mass is equivocal	American College of Sports Medicine, 2009; Garber et al., 2011; Schoenfeld et al., 2016, 2021; Fisher et al., 2017; Krzysztofik et al., 2019; Benito et al., 2020; Travis et al., 2020
**High-intensity RET**	Has been recommended as the preeminent means for enhancing muscle mass and strength due to its common physiological effects, which include an augmentation of muscle capillarization, reduced muscle lipid content, increased motor unit recruitment, and a selective preferential increase in type II muscle fiber recruitment and size that are elicited by this specific stimulus	While proven that it can be done safely and without indices of injury, high-intensity RET may be cautioned against being implemented in specific sectors of the population, such as novice lifters; overreaching may occur as a consequence to high intensity RET if done frequently without adequate recovery time	Fry, 2004; American College of Sports Medicine, 2009; Garber et al., 2011; Fisher et al., 2017; Rader et al., 2018a; Abe et al., 2020; Travis et al., 2020; Vann et al., 2020; Duchateau et al., 2021

## MQ and High-Intensity RET in Young Populations

Despite the critical importance of MQ in meeting the requirements of physically demanding tasks, a comprehensive summary of the effects of enhanced MQ associated with specific exercise regimens has not focused on younger populations in highly physically demanding occupations, such as the military. Synthesizing this information is highly relevant for making evidence-based recommendations for improving MQ in younger populations who regularly engage in physical tasks where optimizing muscle function is of high importance. Before providing a review of studies, it is important to note that the classification of what is appropriately labeled “high-intensity” appears to be quite ambiguous and often differs between researchers (Steele, 2014; Steele et al., 2017). However, there is a general consensus that, when relating intensity to load, ≥70% of 1RM is the minimum threshold for being considered high-intensity (Kristensen and Franklyn-Miller, 2012; Steele, 2014). Therefore, we considered and discussed studies that directly measured MQ where intensity as it relates to load was exclusively or predominantly at ≥ 70% or greater 1RM, while recognizing the limitation of the lack of clarity on a consensus definition. For this review, only studies that utilized SSCs were considered, while those employing isometric, concentric-, or eccentric-only training were excluded given the advantages of this contraction sequence. In addition to these standards, other criteria for inclusion were training studies with a minimum duration of 4 weeks, a minimum training frequency of two times per week, and the requirement that there was at least one metric of skeletal muscle performance/strength and/or muscle mass reported in the study.

Animal-based rodent models have provided an important and fundamental approach to studying MQ responses in young muscle because of the capability to tightly control high-intensity RET paradigms and precisely assess components of MQ. As shown in Table 4, young rats have consistently and robustly responded to an *in vivo* dynamometer model of SSC-loading with a training-induced adaptation in the tibialis anterior (TA), a muscle predominantly comprised of type II fibers (>90%) (Delp and Pette, 1994). This adaptive response is reflected by a ∼15–100% increase in isometric and dynamic skeletal muscle performance as well as a roughly ∼10–25% increase in TA muscle wet-weight, depending on the specific metric utilized and selected method of quantification, ultimately resulting in a robust enhancement of MQ. Additionally, young mice subjected to the same model and training protocol to the plantar flexor muscles responded with a ∼20% increase in dynamic skeletal muscle performance, but with no change in plantaris muscle mass. The other primary model included in Table 4 was a recently developed custom-built voluntary weightlifting apparatus designed for mice to perform squat-like activities against an adjustable load during feeding (Cui et al., 2020). Using this model, substantial increases in plantar flexor contractile force, muscle CSA, and MQ (plantar flexor torque forces normalized to muscle mass) were observed.

**TABLE 4:** Effects of high-intensity RET on MQ in young rodent animal models.

Study	Age/sex/strain	Training model	Study design	Muscle performance^*a*^	Muscle hypertrophy^*a*^	MQ^*a*^	Other notes
Cutlip et al. (2006)	3 month old male FBN rats (*n* = 6)	*In vivo* custom-built rodent dynamometer using electrical stimulation	Maximal RET of the dorsiflexor muscles 3 days/wk for 4.5 wks., 8 sets of 10 SSCs (80 total), 2 min rest between sets	↑ pre-isometric test peak force (25.0%), peak eccentric force (27.9%), negative work (27.0%), and positive work (26.5%) final vs. initial	↑ TA muscle mass normalized to BW (∼33.0%)^*b*^ loaded vs. NLC muscles	↑ TA pre-isometric force normalized to muscle wet mass (0.017 ± 0.001 N/mg loaded muscle	
Baker et al. (2010)	3 month old male FBN rats (*n* = 16)	Same as Cutlip et al. (2006)	Same as Cutlip et al. (2006)	↑ dorsiflexor muscle peak force (∼17.0%)^*b*^ and negative work (∼13.9%)^*b*^ production post- vs. pre-training	↑ TA muscle mass normalized to tibia length (∼16.5%)^*b*^ loaded vs. NLC muscles	↑ TA pre-isometric force normalized to muscle wet mass in loaded (0.015 ± 0.0001 N/mg)	
Rader et al. (2016)	3 and 6 month old male FBN rats (*n* = 5 per group)	Same as Cutlip et al. (2006)	Same as Cutlip et al. (2006)	↑ maximum isometric force and dynamic peak force by (22.0% and 27.0%, respectively) for 3 month old rats final vs. initial; ↔ peak force and positive work capacity ↓ by 27.0% for 6 month old rats final vs. initial	↑ TA muscle mass normalized to tibia length for 3 (20.0%) and 6 month old (16.0%) rats loaded vs. NLC muscle	↑ TA isometric force divided by normalized muscle mass in loaded for both 3 (0.64 N/mg/mm) and 6 month old (0.63 N/mg/mm)^*b*^ rats	↑ (11.0–15.0%) fatigue recovery for peak force, negative work, and positive work at final vs. initial in 6 month old rats
Rader et al. (2017b)	3 month old male FBN rats (*n* = 4–8)	Same as Cutlip et al. (2006)	Same as Cutlip et al. (2006)	↑ (∼24.0–27.0%)^*b*^ peak force output for rats at both frequencies final vs. initial; ↑ (∼15–22%)^*b*^ negative work at both frequencies final vs. initial	↑ (19.0–20.0%) in TA muscle mass normalized to tibia length at both frequencies loaded vs. NLC muscle	↑ final maximum isometric force divided by normalized muscle mass at both frequencies (0.59–0.65 N/mg/mm)^*b*^	No differences for MQ between 2 or 3 day/wk training; chi-square analysis of muscle mass fiber size area distributions showed an ↑ at both frequencies
Rader et al. (2018a)	3 month old male FBN rats (*n* = 9–10 per group)	Same as Cutlip et al. (2006)	Maximal RET of the dorsiflexor muscles 3 days/wk. for 4.5 wks., 4 sets of one isometric repetition (4 total) or 4 sets of 10 SSCs (40 total), 2 min rest between sets	↑ peak force at final vs. initial for both 4 isometric contractions (20.1%) or 40 SSCs (16.5%); ↑ isometric peak force at final vs. initial for both 4 isometric contractions (13.9%) or 40 SSCs (8.0%; p = 0.097)	↑ TA muscle mass normalized to tibia length in loaded vs. NLC muscle for both 4 isometric contractions (7.0 ± 2.0%) and 40 SSCs (17.0% ± 2.0%); ↑ (∼8.3%)^*b*^ TA muscle mass normalized to tibia length of the loaded muscles of 40 SSCs vs. 4 isometric contractions	↑ maximum isometric force divided by normalized muscle mass (i.e., muscle wet weight divided by tibia length) in loaded TA of both 40 SSCs and 4 isometric contractions (0.58 ± 0.03 N/mg/mm and 0.65 ± 0.02 N/mg/mm, respectively)	
Rader et al. (2018b)	3 month old male Snell dwarf mice and age-matched normal-sized littermate CONs^c^	*In vivo* dual mode muscle lever system that allows measurement of torque and control of foot rotation about the ankle	Maximal training of the plantar flexor muscles 3 days/wk. for 4.5 wks., 8 sets of 10 SSCs (80 total), 2 min rest between sets;	↑ (20.0%) plantarflexion peak muscle torque and maximum isometric torque in loaded vs. NLC muscle	↔ in GTN normalized muscle mass (i.e., muscle wet weight divided by tibia length) or normalized muscle mass per gram of BW	↑ (21.4%)^*b*^ maximal isometric torque divided by normalized muscle mass in loaded vs. NLC muscle (1.4 vs. 1.7 mN-m/mg/mm, respectively)^&^	
Cui et al. (2020)	10–14 wk. old male C57BL/6J mice	Voluntary squat-like weightlifting activity against an adjustable load in cages where during feeding,	Mice trained hindlimb muscles 5 days/wk. for 8 weeks; load relative intensity started at 100.0% of BW on the first day, then incrementally went up 20.0% each day until reaching 240.0% of their BW load.	↑ plantar flexor muscle contractile function properties measured during twitch contraction, including contraction speed (1.33 fold), relaxation speed (1.34 fold), and integrated work (1.37 fold) weightlifting vs. CON mice	↑ in hindlimb muscle CSA normalized to BW (17.0%), measured by MRI, and GTN muscle wet mass (14.0%) weightlifting vs. CON	↑ (35.7%) plantar flexor twitch torque normalized to muscle mass weightlifting vs. CON (0.19 ± 0.01 mN*m/mg vs. 0.14 ± 0.01 mN*m/mg, respectively); ↑ (19.8%) plantar flexor tetanic torque normalized to muscle mass weightlifting vs. CON (1.15 ± 0.03 mN*m/mg vs. 0.96 ± 0.02 mN*m/mg, respectively)	

*^*a*^Results significant at a *p* < 0.05 level except where noted directly in text.*

*^*b*^Estimated value(s) based on contained figure(s) within manuscript.*

*^*c*^Only normal-sized littermate CONs with no genetic mutations are reported ↑, increase; ↓, decrease; ↔, no change. MQ, muscle quality; FBN, Fischer 344 x Brown Norway; SSC, stretch-shortening contraction; RET, resistance exercise training; TA, tibialis anterior; BW, body weight; NLC, non-loaded control muscle; CSA, cross-sectional area; CON, control; GTN, gastrocnemius; MRI, magnetic resonance imaging.*

Besides the summary of the results for MQ provided in Table 4, a key observation regarding fiber type distribution has been that young rats (3 month old) have demonstrated a transition to a more oxidative phenotype with high-intensity RET, either from type IIb to IIx (Rader et al., 2016, 2017a) or type IIx to IIa (Aguiar et al., 2013). Also, with respect to adult skeletal muscle, results from a study (Rader et al., 2016) indicated there was an altered phenotype occurring prior to complete biological development of rodents, which generally occurs by 6 months of age, or young adulthood. This was highlighted by impairments in the adaptive capacity due to a blunted MQ response following the same high-intensity RET protocol in comparison to 3-month-old rats who had markedly increased MQ (Cooper et al., 2015; Rader et al., 2016). These results indicate that skeletal muscle can potentially experience declines in its ability to adapt with age, including at the onset of young adulthood. These outcomes, should they hold true for humans, have important implications for young adult military personnel who are still expected to perform at high levels of function during physically demanding tasks.

The effects of high-intensity RET (>70% 1RM) on MQ, as defined by muscle strength relative to mass, has been investigated in young, healthy human populations (Table 5). Collectively, following high-intensity RET, all but one of these studies showed significant improvements in muscle strength/performance (∼4–75%), and all seven studies showed increases in muscle mass (∼4–35%). Ultimately, these studies had an overall ∼8.5–35.0% increase in MQ following RET. Of these studies, four used untrained participants (Cureton et al., 1988; Chestnut and Docherty, 1999; Ivey et al., 2000) and in two others, training status was not reported (Dons et al., 1979; Welle et al., 1996). It is important to acknowledge that untrained individuals respond differently to RET than trained individuals. In the reviewed studies, untrained individuals respond positively to the vast majority of RET programs, and thus specific nuances to the exercise prescription are less critical (Aagaard and Andersen, 1998). Castro et al. (1995) found trained participants had significantly greater mean torque per unit of muscle and bone CSA for the upper arm (28.9%) and thigh (18.8%) compared to untrained participants following RET (Castro et al., 1995), indicating that trained participants potentially have greater capabilities to augment MQ in response to high-intensity RET. Two of the aforementioned studies made direct comparisons between different RET intensities. One study found MQ to be 30% higher at post-training in a group that trained at 80% 1RM, whereas there was only a 5% increase in post-training MQ in a 50% 1RM group (Dons et al., 1979). In the other study, there were no changes in MQ between 4RM vs. 10RM group (Cureton et al., 1988). However, since both groups trained at relatively high intensities of 85% and 70% 1RM, respectively, along with the lack of other available direct comparisons, it is difficult to draw any conclusions on the effects of specific intensities on MQ in young populations following high-intensity RET.

**TABLE 5:** Effects of high-intensity RET on MQ, as measured by relative strength, in young (18–50 years), healthy humans.

Study	Population	Study design	Muscle performance^*a*^	Muscle hypertrophy^*a*^	MQ^*a*^	Other notes
Cureton et al. (1988)	22 UT males and females (22–37 years); 15 (7 males, 8 females) took part in RET program	Individualized progressive full-body heavy FW and machine RET program 3 days/wk for 16 wks; 1–3 sets performing as many repetitions as possible to failure using flexion and extension movements of the arms and legs at 70.0–90.0% of 1RM. Absolute and relative intensity were progressively increased from an initial 70.0% to 80.0–90.0% of 1RM toward the end of the study	↑ elbow flexion (36.2%), elbow extension (32.6%), knee flexion (12.8%), and knee extension (28.8%) strength trained vs. CON males post-training; ↑ elbow flexion (59.2%), elbow extension (41.7%), knee flexion (24.4%), and knee extension (33.9%) strength trained vs. CON females post-training	↑ Bone plus muscle CSA estimated by anthropometry (17.5%), and CT scan muscle CSA (15.9%) trained vs. CON males; ↑ Bone plus muscle CSA estimated by anthropometry (20.4%), and CT scan muscle CSA (22.8%) trained vs. CON females; ↔ change in bone plus muscle CSA estimated by anthropometry, and CT scan muscle CSA trained vs. non-trained CON in males and females	↑ mean ratio elbow flexor + elbow extensor strength to mean upper arm CSA in males (16.2%) and females (22.5%); ↑ mean ratio knee flexor + knee extensor strength to mean leg CSA in males (19.6%) and females (25.0%) at post-training	Similar MQ response between males and females; absolute or average number of repetitions performed until failure during training and overall training volume not reported
Kawakami et al. (1995)	5 healthy males (29.0 ± 4 y); volunteers were “well-accustomed to the development of maximal voluntary force”	Unilateral RET of the elbow extensor muscles 3 days/wk for 16 wks at 80% 1RM.	↑ isokinetic dynamometer isometric (16.0%) and isokinetic (20.0–32.0% concentric, 15.0–16.0% eccentric) peak torque of the elbow extensors in trained post- vs. pre-training	↑ MRI muscle volume (31.7%), anatomical CSA (31.7%), physiological CSA (33.3%), and muscle layer thickness (27.0%) of TB post- vs. pre-training;	↓ (∼8.5–28.3%)^*b*^ specific tension (torque output divided by physiological CSA) post-vs. pre-training	Comparisons for MQ between trained vs. non-trained CON arm not reported; rest interval between sets for training not provided
Castro et al. (1995)	26 males and females (18–30 years; half previously T, half UT	T group engaged in heavy RET for at least 1 year; training program for study consisted of full-body exercises 2–3 days/wk6–10 sets of 4–10 repetitions (∼75.0–90.0% 1RM)^*c*^	↑ upper arm (75.4%) and thigh torque (40.2%) T vs. UT males; ↑ in upper arm torque (39.9%) and thigh torque (29.5%) T vs. UT females post-training	↑ in upper arm (35.0%) and thigh (18.9%) muscle and bone CSA T vs. UT males post-training; ↔ upper arm or thigh muscle and bone CSA T vs. UT females post-training	↑ Mean torque per unit muscle and bone CSA for the upper arm (28.9%) and thigh (18.8%) T vs. UT at post-training regardless of gender; ↑ (23.6%) mean upper arm torque/CSA when adjusted for bone T vs. UT at post-training regardless of gender; ↔ female to male ratios of torque/muscle and bone CSA for the upper arm and thigh (0.95–1.01)	When torque was expressed per unit average BW or LBM, large gender differences for the upper arm remained, whereas the thigh was similar; type of RET (e.g., FW, machines), specific exercises selected, and training duration were not reported
Welle et al. (1996)	Males and females (22–31 years)	Full-body training performed on machines 3 days/wk for 3 months, 3 sets of 8 repetitions (∼80.0% 1RM)^*c*^	↔ mean ratio of training weight to 3RM ratio of the elbow flexors, knee flexors, and knee extensors post- vs. pre-training	↑ MRI muscle CSA of the elbow flexors (22.2%), knee flexors (7.9%), and knee extensors (3.6%) at post- vs. pre-training	↑ specific tension (ratio of 3RM strength to maximum CSA) of the elbow flexors (21.0%), knee flexors (28.3%), and knee extensors (38.1%) at post- vs. pre-training	Training status prior to start of study not provided; gender comparisons for MQ were not reported
Chestnut and Docherty (1999)	24 UT males (24.2 ± 1.8 y)	Upper-body FW RET performed 3 days/wk. for 10 wks.; participants were assigned to a 4RM or 10RM group. 4RM group performed 6 sets of 4 repetitions at ∼85% 1RM with 3 min rest intervals, while 10RM group performed 3 sets of 10 repetitions at ∼70% 1RM with 2 min rest	↑ 1RM in both 4RM and 10RM groups for forearm extensor strength at pre- vs. mid-training (25.0% and 10.0%, respectively)^*b*^ and mid- vs. post-training (9.4% and 7.8%, respectively)^*b*^; ↑ 1RM in both the 4RM and 10RM groups for flexed forearm strength pre- vs. mid-training (6.8% and 7.0%, respectively)^*b*^ mid- vs. post-training (10.6% and 4.3%, respectively)^*b*^; ↔ non-exercise CON	↑ MRI CSA in both the 4RM and 10RM groups at for the forearm extensor and flexor muscle groups at both the midpoint (10.4% and 9.2%, respectively)^*b*^ and distal (8.3% and 7.4%, respectively) axial scan regions at post- vs. pre-training; ↔ non-exercise CON	↑ specific tension (combined 1RM tricep press and barbell curl scores divided by MRI CSA) in both the 4RM and 10RM groups at both the midpoint (10.0% and 6.5%, respectively) and distal (9.8 and 7.5%, respectively) axial scan regions at post-vs. pre-training; ↔ non-exercise CON	No changes MQ detected between 4RM vs. 10RM; no statistical comparisons reported between training vs. non-exercise CON
Ivey et al. (2000)	Healthy UT males and females (20–30 years)	Dominant leg knee extension 3 days/wk for 9 wks; after warm-up, 4 sets using 5RM values (∼87.0% 1RM)^*c*^ performed at near maximal effort for total of 10–15 repetitions per set	↑ 1RM quadriceps strength in males (25.6%) and females (40.3%) post- vs. pre-training	↑ MRI quadriceps muscle volume in males (12.1%) and females (5.5%) post- vs. pre-training	↑ quadriceps 1RM/muscle volume ratio in males (∼11%)^*b*^ and females (∼35.0%)^*b*^ post- vs. pre-training	Females had greater increases in MQ vs. males
Yasuda et al. (2011)	Recreationally active UT males (22–32 years)	Supervised FW bench press 3 days/wk. for 6 wks., 3 sets of 10 repetitions 75% 1RM, 2–3 min rest between sets	↑ bench press 1RM (17.5%)^*c*^ and maximum isometric strength of the elbow flexors (26.9%) post- vs. pre-training	↑ (8.6%) MRI TB CSA post- vs. pre-training	↑ (3.3%) relative isometric strength (maximum voluntary contraction divided by TB CSA) post- vs. pre-training; ↑ (10.5%) relative dynamic strength (1RM divided by TB CSA) post- vs. pre-training; ↑ relative isometric strength post-training compared to both low-intensity blood flow restriction training and non-exercised CON (3.3% vs. –3.5%, vs. –0.1, respectively)	12 of 40 participants engaged in regular aerobic-type exercise (walking, jogging, or cycling 2–3 times/wk for ∼30 min); 9 participants had light to moderate RET experience performing bench presses

*Studies included are those that recruited younger adults (18–50 years), used stretch-shortening contraction resistance exercise training at a frequency of ≥ 2x/week for at least 4 weeks at a minimum intensity of 70% 1RM.*

*^*a*^Most relevant performance metric(s) extracted from these studies when multiple measures (ex: peak power, mean power) were presented.*

*^*b*^Estimated value(s) based on contained figure(s) within manuscript.*

*^*c*^1RM values based off NSCA guidelines that have defined the percent 1RM-repetition relationship (Baechle and Earle, 2008). ↑, increase; ↓, decrease; ↔, no change. MQ, muscle quality; UT, untrained; RET, resistance exercise training; FW, free weight; RM, repetition maximum; CON, control; CSA, cross-sectional area; CT, computerized tomography; MRI, magnetic resonance imaging; TB, triceps brachii; T, trained; BW, body weight; LBM, lean body mass.*

Given that published research provides support that high-intensity RET improves muscle strength, mass, and composition in young individuals, proxy measures of MQ assessed with imaging techniques such as EI measured via US would also be expected to demonstrate this MQ improvement. However, a majority of the research demonstrating that exercise interventions increase MQ as reflected by a decrease in muscle EI have been conducted in older adults or diseased populations (Radaelli et al., 2013, 2014; Cadore et al., 2014; Wilhelm et al., 2014; Yoshiko et al., 2017, 2018; Yamada et al., 2019). Few investigations have examined the impact of RET on EI in young individuals, making it difficult to make definitive conclusions. Nevertheless, studies examining the effects of high-intensity RET on EI in younger populations listed in Table 6 demonstrate comparable training-induced improvements in muscle performance and/or mass to those in Table 5. Furthermore, the majority of these studies showed enhanced MQ (i.e., decreased EI of ∼5–16%) following high-intensity RET. However, two of the aforementioned studies, despite showing increased muscle performance (∼4–22%) and size (∼3–7%), did not report changes in MQ (Wells et al., 2014; Jenkins et al., 2016). A number of factors related to US measurement could have affected the results, such as probe placement, muscle depth, subcutaneous adipose tissue thickness, and patient positioning (Young et al., 2015; Dankel et al., 2018; Varanoske et al., 2019). Of these studies, three performed direct comparisons between high and low intensity training and showed that high-intensity training resulted in greater post-training relative increases in muscle performance and hypertrophy, with two of the three also reporting greater decreases in EI (higher improvements in MQ) in the high-intensity group (Jenkins et al., 2017; Ikezoe et al., 2020). Taken together, these findings demonstrate that RET in young individuals may improve MQ as assessed via ultrasonography.

**TABLE 6:** Effects of high-intensity RET on MQ measured via ultrasound-derived EI in young (18–50 years) humans.

Study	Population	Study design/Training program	Muscle performance^*a*^	Muscle hypertrophy^*a*^	MQ^*a*^	Other notes
Jajtner et al. (2013)	28 Division I female soccer players (20.5 ± 1.2 y)	Full-body RET 2 days/wk for 12 wks; 3 sets of 6–8 repetitions (∼80.0–85.0% 1RM)^*c*^ except high pull at 4–6 reps (∼85.0–90.0% 1RM)^*c*^ and squat jump at 3 reps (∼93% 1RM)^*c*^	↔ vertical jump peak power	↑ VL CSA (starters: 2.5%; non-starters: 3.30%); ↔ in RF CSA or VL MT and CSA	↓ RF EI for starters (7.3%) and non-starters (5.8%); ↔ VL EI	↓ VL PA (1.5–12.5%); from pre to post; ↔ in RF PA; rest interval between sets not reported
Jenkins et al. (2016)	15 UT, healthy males (21.7 ± 2.4 y)	Forearm flexion RET to failure 3 days/wk (3 sets) for 4 wks; high load (80% of 1RM) or low load (30% of 1RM) groups	↑ post-training forearm flexion 1RM in high load (21.3%), ↔ in low load; ↑ forearm flexion maximum voluntary isometric strength in high load (21.8%), ↔ in low load	↑ post-training forearm flexor MT, collapsed across location (high load: 6.5%; low load: 7.4%)^*b*^;	↔ forearm flexor EI in either group	
Jenkins et al. (2017)	26 UT, healthy males (23.1 ± 4.7 y)	Leg extension RET to failure 3 days/wk for 6 wks; high load (80% 1RM) or low load (30% 1RM) groups	↑ post-training leg extension 1RM, high- vs low load (∼30.6%; vs. ∼8.6%)^*b*^; ↑ post-training leg extension maximum voluntary isometric strength high vs. low load (∼28.6% vs. ∼7.7%)^*b*^	↑ post-training VL MT in both groups (high load: ∼7.3%; low load: ∼4.4%)^*b*^ post- vs. pre-training	↓ post-training VL EI in high load (∼7.8%)^*b*^ as well as a trend (p = 0.07) for low load (∼5.2%)^*b*^	
Mangine et al. (2018)	15 physically active, resistance-trained males (24.0 ± 3.0 y)	4 lower-body exercises 2 days/wk for 8 wks; 4 sets 3–5 reps (90% 1RM), 3 min rest	None reported	↑ CSA (RF and VL combined: 7.7%); greater increases in VL than RF CSA; ↔ in MT	↓ post-training proximal VL EI (∼6.4%)^*b*^; ↔ RF EI	Trend for ↑ in PA (RF and VL combined: 5.3%; *p* < 0.10)
Ikezoe et al. (2020)	15 UT, healthy males (23.1 ± 2.6 y)	Leg extension RET on dynamometer 3 days/wk for 8 wks; high load (80% of 1RM) or low load (30% of 1RM) groups	↑ leg extension 1RM in both groups (high load: 40.9%; low load: 36.2%); ↑ leg extension maximum voluntary isometric strength in both groups (high load: 25.5%; low load: 24.0%)	↑ VL MT in both groups (high load: 20.4%; low load: 11.3%)	↓ VL EI in both groups (high load: 16.3%; low load: 8.1%)	↔ post-training between high vs. low load for MQ
Stratton et al. (2020)	26 recreationally active males (∼22.9 year)	Full body FW RET 3 days/wk for 4 wks; intensity for bench press/leg press ranged from 70–87.5% 1RM, other exercises based off reps in reserve; 2–4 sets, 3–8 reps,;∼2 min rest between sets	↑ 1RM bench press (5.3–6.9%)^*b*^ and leg press (8.1–10.6%)^*b*^, post- vs. pre-training	↑ VL CSA (4.2–8.7%) and BB (5.6–6.7%) post- vs. pre-training; ↑ BB MT (13.0%) post- vs. pre-training; ↔ VL or RF MT or RF CSA	↓ VL EI (5.3–7.5%) post vs. pre-training; ↔ RF or BB EI	No differences in MQ between time-restricted feeding vs. normal diet; time-restricted feeding group had 25% energy deficit
Wells et al. (2014)	23 Division I female soccer players (19.7 ± 1.0 y)	Whole-body RET 4 days/wk for 15 wks; 2–4 sets, 3–10 repetitions (∼75–93% 1RM)^*c*^ except one exercise (see notes)	↑ post-training 1RM squat (4.2%)	↑ post-training VL CSA (2.6%); ↑ VL MT at medial location (9.5%)	↔ VL EI	↔ VL PA at proximal or medial locations; glute-ham raises performed at 12–15 repetitions (∼65–67%) 1RM for first 2 wks, but subsequently were performed at ≥ 70% for final 13 wks

*Studies included are those that recruited younger adults (18–50 years), used stretch-shortening contraction resistance exercise training at a frequency of ≥2x/week for at least 4 weeks at a minimum intensity of 70% 1RM, and reported ultrasound-derived EI values.*

*^*a*^Most relevant performance metric(s) were extracted from these studies when multiple measures (ex, peak power, mean power) from were presented.*

*^*b*^Estimated value(s) based on contained figure(s) within the manuscript.*

*^*c*^1RM values are based off NSCA guidelines that have defined the percent 1RM-repetition relationship (Baechle and Earle, 2008); ↑, increase; ↓, decrease; ↔, no change. MQ, muscle quality; RET, resistance exercise training; RM, repetition maximum; VL, vastus lateralis; CSA, cross-sectional area; RF, rectus femoris; EI, echo intensity; UT, untrained; BB, biceps brachii; MT, muscle thickness; PA, pennation angle.*

Additional evidence demonstrates that the components of muscle adaptation that MQ is derived from (i.e., force to size ratio) is noticeably improved by high-intensity RET. In a recent review by Abe et al. (2020), the authors found from RET studies using heavy exercise loads (>70% 1RM) increased vastus lateralis muscle CSA (∼11%), pennation angle (∼6%), and fascicle length (5%). Aube et al. (2020) used young well-trained male participants to investigate the effects of three different 8-week high-intensity lower-body progressive RET interventions (loads equivalent to ∼80–85% 1RM) differing in the number of total sets performed. All three groups showed significant improvements in back squat 1RM muscle strength ranging from ∼5.4 to 16.2%; additionally, all three groups demonstrated a significant increase in thigh muscle thickness (∼6.1–7.7%). In another study by Lasevicius and colleagues (Lasevicius et al., 2018), the authors found following 12 weeks RET that the highest intensity group (80% 1RM) demonstrated superior increases in muscle strength and CSA in comparison to low intensities. Mangine et al. (2015b) found in response to their 8 week moderate or high-intensity RET program, both groups responded with significant increases in bench press and back squat 1RM strength; however, the high-intensity group that trained at 90% of 1RM also had significantly higher bench press 1RM compared to the moderate group.

However, not all studies have shown positive effects of high-intensity training on MQ. For example, a previous investigation used data questionnaires in college-aged males and females, and based on answers about previous training history, assigned individuals to either a low- or high-intensity resistance training group and subsequently performed a battery of tests looking at muscle function (Shoepe et al., 2020). The high-intensity group had significantly greater handgrip strength but both groups had lower MQ compared to individuals with no history of RET. However, there were several limitations; the study design was retrospective in nature as the authors relied on participant questionnaire responses for group assignments, so an inherent self-bias could have led to inaccurate answers and group placement. Additionally, due to the retrospective design, the exercise intervention conducted was not under the supervision of the investigators; previous research has shown larger effects in strength when RET is supervised (Mazzetti et al., 2000; Lacroix et al., 2017). Finally, the authors chose handgrip strength as a surrogate measure of total body strength. Previous research has shown that handgrip strength is not the best indicator of physical performance (Abe et al., 2016) and that applying 1RM would have been a more representative outcome metric in calculating MQ. In another study by Nóbrega and colleagues (Nobrega et al., 2018), young men (average age: 23.0 years old) participated in two days per week of unilateral leg extension RET for 12 weeks at 30% and 80% 1RM. Following both 6 (midpoint) and 12 weeks (post-training) of exercise, similar improvements in 1RM muscle strength, muscle CSA, and pennation angle were observed. However, training was done using a unilateral leg extension machine (3 sets total per training session), so perhaps the magnitude of the results in the high-intensity groups were hampered by the fact that participants were not performing full body RET with free weights and with overall low session training volume. As noted earlier, a benefit of high-intensity RET is the ability to maximally recruit the majority or all the fibers of a targeted muscle group (Rader et al., 2017b), which is somewhat offset using machines. Although some previous research has demonstrated that short-term (8-week) RET training with free weights or machines induces similar increases in muscle mass and strength (Schwanbeck et al., 2020), free weight usage has resulted in greater electromyography muscle activation (McCaw and Friday, 1994; Schwanbeck et al., 2009), and greater increases in circulating testosterone concentrations (Schwanbeck et al., 2020) than RET using machines. As most improvements in muscle strength during the first phases of training are due to neural adaptations rather than muscle hypertrophy (Moritani and deVries, 1979; Sale, 1988), it is not surprising that increases in muscle mass during short-term RET are similar with free weights and machines (Schwanbeck et al., 2020). However, the greater anabolic response and neural activation observed with free weight RET provide promising evidence that increases in strength and/or hypertrophy may be greater when using free weight RET than machines during longer-term training regimens. Collectively, these studies provide evidence strongly supporting the notion that high-intensity RET, in comparison to other exercise-based interventions, is the most effective exercise strategy for the augmentation of force production, relevant muscle mass, power development, and, ultimately, MQ.

## Conclusion

While most practical measurement techniques will not capture all of the complex adaptations of muscle to an exercise training stimulus, measurements that account for MQ ultimately reflect the functional summation of complex physiologic changes in response to adaptations to training. MQ has historically been evaluated in older adult and/or clinical populations. However, the paradigm shift observed in the field of aging, to include a focus on MQ rather than a mere loss of muscle mass, has not been replicated to the same degree in young populations. Thus, we reviewed the importance of this measurement in young, healthy adults and highlighted studies that have emphasized the importance of MQ in this cohort.

The collective results from the literature in animals and humans that have utilized physiologically relevant exercise models indicate that high-intensity RET, compared to other exercise training regimens, has the most robust positive effects on augmenting MQ. Younger individuals engaged in intense physical tasks do not simply need to acquire large amounts of muscle mass, but would benefit from increased functional capacity in order to successfully complete their duties. Hence, we put forth the assertion that MQ should be recognized as one of the most important biomarkers for performance enhancement in young individuals. Accordingly, focusing on evidence-based exercise interventions that serve to further enhance MQ in various young populations, such as high-intensity RET, will ultimately improve the capabilities of these individuals to optimally perform the critical responsibilities related to their physically demanding occupational tasks.

## Author Contributions

All authors listed have made a substantial, direct and intellectual contribution to the work, and approved it for publication.

## Author Disclaimer

The opinions or assertions contained herein are the private views of the authors and are not to be construed as official or as reflecting the views of the Army or the Department of Defense.

## Conflict of Interest

The authors declare that the research was conducted in the absence of any commercial or financial relationships that could be construed as a potential conflict of interest.

## Publisher’s Note

All claims expressed in this article are solely those of the authors and do not necessarily represent those of their affiliated organizations, or those of the publisher, the editors and the reviewers. Any product that may be evaluated in this article, or claim that may be made by its manufacturer, is not guaranteed or endorsed by the publisher.
